# Sp1 transcription factor represses transcription of phosphatase and tensin homolog to aggravate lung injury in mice with type 2 diabetes mellitus-pulmonary tuberculosis

**DOI:** 10.1080/21655979.2022.2062196

**Published:** 2022-04-14

**Authors:** Hongmei Zhao, Lian Shi, Xiaohong Wang, Xiuli Yu, Danfeng Wang

**Affiliations:** a Department of Tuberculosis, Shenyang Chest Hospital, Shenyang, Liaoning, China; b Department of Respiratory and Critical Care, Shenyang Chest Hospital, Shenyang, Liaoning, China

**Keywords:** Type 2 diabetes mellitus, pulmonary tuberculosis, PTEN, SP1, Akt

## Abstract

Type 2 diabetes mellitus (T2DM) can enhance the risk of mycobacterium tuberculosis (Mtb) infection and aggravate pulmonary tuberculosis (PTB). This study intended to explore the function of phosphatase and tensin homolog (PTEN) in T2DM-PTB and the molecules involved. Mice were treated with streptozotocin to induce T2DM and then infected with Mtb. The mice with T2DM had increased weight, blood glucose level, glucose intolerance and insulin resistance, and increased susceptibility to PTB after Mtb infection. PTEN was significantly downregulated in mice with T2DM-PTB and it had specific predictive value in patients. Overexpression of PTEN improved mouse survival and reduced bacterial load, inflammatory infiltration, cell apoptosis, and fibrosis in lung tissues. Sp1 transcription factor (SP1) was predicted and identified as an upstream regulator of PTEN. SP1 suppressed PTEN transcription. Silencing of SP1 enhanced mouse survival and alleviated the lung injury, and it promoted the M1 polarization of macrophages in murine lung tissues. However, further downregulation of PTEN increased protein kinase B (Akt) phosphorylation and blocked the alleviating roles of SP1 silencing in T2DM-PTB. This study demonstrates that SP1 represses PTEN transcription to promote lung injury in mice with T2DM-PTB through Akt activation.

## Graphical abstract

**Figure:**
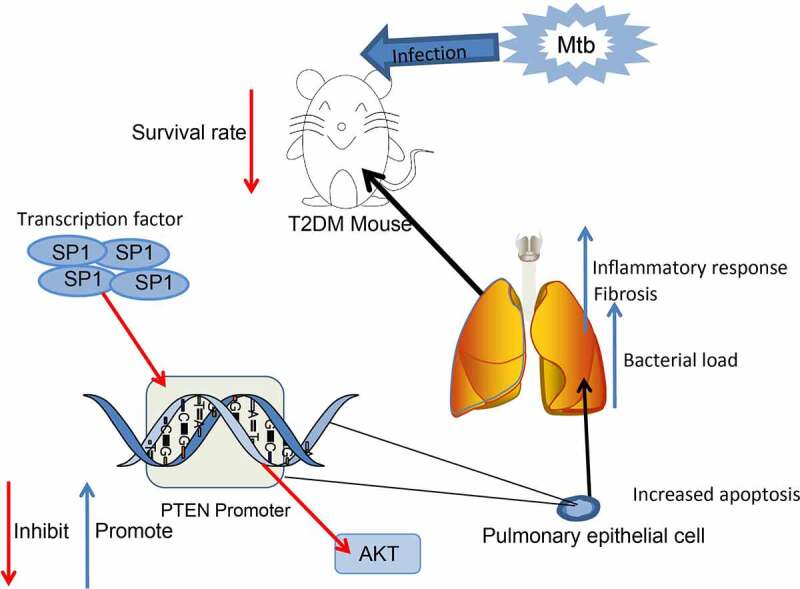

## Introduction

Tuberculosis (TB) is a leading cause of death from a single infectious disease agent mycobacterium tuberculosis (Mtb), and over 10 million people develop active disease worldwide annually, predominately active pulmonary TB (PTB) [1–3]. Type 2 diabetes mellitus (T2DM) has been well-established as a major risk factor of TB, and patients with DM were found to have higher risk for development of active TB by 2 to 8 folds than those without [4–6]. DM is also correlated with greater severity of TB disease, influencing both disease symptoms and the treatment efficacy, leading to increased risk of treatment failures and relapse, and the TB might in turn induce glucose intolerance and worse glycemic control in patients with DM [4,7,8]. The first-line regimen for drug-sensitive TB includes isoniazid, rifampicin, pyrazinamide and ethambutol for over 6 months of treatments [9]. Given that the incidence of DM is soaring, it is urgent to identify key molecular mechanisms involved in the development of T2DM-assoicated PTB (T2DM-PTB) to develop novel strategies for the disease control.

Macrophages are the primary targets of Mtb infection [10]. During Mtb infection, both alveolar and interstitial macrophages modulate immune responses and play key roles in defending against infection [11]. The significant diversity and plasticity of macrophage functions are determined by their polarization state of M1 (classically activated macrophages) or M2 (alternatively activated macrophages) [12,13]. During the early stages of infections, infected macrophages induce differentiation of T helper type 1 (Th1) cells; resulting in elevated production of Th1 and Th1-like cytokines which play crucial roles in the establishment of antimycobacterial host resistance; however, in the advanced stages, host immune systems are likely to adopt a Th2-type immune response via the induction of Th2 cells that diminish the antimycobacterial cellular immunity [14,15]. Therefore, the early interaction between macrophage polarization and Mtb largely determine the subsequent progression of infection.

Phosphatase and tensin homolog (PTEN) is a well-known tumor suppressor via its suppressive function in cell survival-associated phosphatidylinositol 3-kinase/protein kinase B (PI3K/Akt) signaling pathway [16–18]. One of the key downstream effectors of the pathway, the mammalian target of rapamycin (mTOR)-Raptor kinase complex, is a master regulator of autophagy and cellular metabolism [19]. Therefore, genetic alteration of PTEN or other components of this pathway may lead to the pathogenesis of cancers or other diseases. Suppression of the PI3K/AKT signaling and the resultant activation of autophagy has been reported to be involved in Mtb clearance in macrophages [20]. Importantly, PTEN has been reported with the ability in inhibiting mycobacterial infection [21]. Intriguingly, PTEN was found to be downregulated in diabetic mice [22]. Therefore, we postulated that PTEN might be aberrantly expressed in T2DM and potentially play roles in Mtb clearance, thus affecting the susceptibility to PTB in T2DM. We established diabetic mice and treated them with Mtb to investigate the relevance of PTEN to Mtb infection and macrophage polarization in T2DM-PTB and to explore the molecules implicated.

## Methods and materials

### Establishment of a murine model of T2DM

C57BL/6 J mice (4 weeks old, 20 ± 5 g) were procured from SJA Laboratory Animal Co., Ltd. (Hunan, China). Streptozotocin (STZ) was dissolved in 50 mmol/L citrate buffer, and 180 mg/kg mixed solution was intraperitoneally injected into mice for three times at a 10-day interval. The mice were intraperitoneally injected with 60 mg/kg nicotinamide dissolved in saline water 15 min prior to the STZ injection [23].

After 8 weeks, the blood glucose level in mice was examined using an Ascensia Elite glucometer (Bayer AG, Leverkusen, Germany), and the mice were weighed to validate whether the murine model of T2DM was successfully established. Mice in the sham group were injected with an equal amount of dimethyl sulfoxide solution. The mice were subjected to glucose tolerance test (GTT) and insulin tolerance test (ITT). For GTT, the mice were starved for 12 h and fed with 1.5 g/kg glucose, and the blood glucose level of mice was examined at the 0, 30, 60, 90 and 120 min. For ITT, the mice were starved for 12 h and treated with 1 U/kg insulin, and the glucose level at the 0, 30, 60, 90 and 120 min was examined as well. Moreover, the blood sample of mice was collected. In short, the mouse was fixed, and both sides of the neck were pressed to promote the hyperemia of orbital venous plexus. A capillary segment soaked in 1% heparin was inserted into the conjunctiva to cut the venous plexus, and the blood flow out along the capillaries was collected. After that, the mouse was bandaged with gauze for hemostasis.

At the 8^th^ week after model establishment, 100 μL lentiviral vectors-carried overexpression vector of PTEN (PTEN-OE) and the negative control (NC) PTEN-NC, small interfering (si) RNA of SP1 (si-SP1) and PTEN (si-PTEN) and the control SP1-NC (all 5 × 10^8^ PFU) (Sangon Biotech Co., Ltd., Shanghai, China) were intraperitoneally injected into mice. For the maintenance of stable expression, the mice were repeatedly administrated with two times of lentiviral vectors at each injection. The injections were performed in one month at a 10-day interval. All animal procedures were approved by the Animal Ethics Committee of Shenyang Chest Hospital (Approval Number: 2020–005-01) and adhered to the Guide for the Care and Use of Laboratory Animals (National Institutes of Health, Bethesda, MD, USA).

### Induction of PTB in mice with T2DM

The mice were infected with Mtb H37Rv (American Type Culture Collection, Manassas, VA, USA) to induce PTB. In short, an aerosol generator/inhalation system (Glas-Col Corp, Terre Haute, IN, USA) was used for aerosol generation. The animals were housed in a box and inhaled with air with H37Rv. The H37Rv was suspended in 0.1% bovine serum albumin-V and 0.1% Tween-80 solution and put into the atomizer. The compressed air was adjusted to 5 L/min, and the main air flow was approximately 28 L/min (1 cfm). After 30 min of atomization, the mice were disinfected and treated with the aerosol [23].

The survival time of mice was set at 200 days. The mice were euthanized before the presence of physiological pain through intraperitoneal injection of 1% pentobarbital sodium (120 mg/kg). The animal death was confirmed by the loss of neural reflex, blink reflex and heartbeats, and the possibility of cardiac arrest was excluded. The lung tissues of all mice were collected. The left lung tissues were cut into slices for histopathological staining, and the right lung tissues were made into homogenate for RNA and protein determination. The tissue homogenate was coated on oleate albumin glucose catalase-contained 7H10 agar plates (HB6270, HopeBio Co., Ltd. Qingdao, Shandong, China). After 22 days of incubation at 37°C for 5% CO_2_, the bacterial load was examined using the colony-forming unit (CFU) analysis.

### Hematoxylin and eosin (HE) staining

The lung tissues were fixed in 4% paraformaldehyde for 48 h, embedded in paraffin, and cut into 4-μm slices for HE staining. The tissues were baked in a 45°C incubator, dewaxed, and rehydrated, and stained with hematoxylin (C0107, Beyotime Biotechnology Co., Ltd., Shanghai, China) for 5 min, differentiated in 1% HCl-ethanol for 3 s, and then stained with 5% eosin (C0109, Beyotime) for 3 min. The slices were then sealed with neutral balsam and observed under the microscope (Olympus Optical Co., Ltd, Tokyo, Japan) [24].

### mRNA microarray analysis

The murine lung tissues were pre-hybridized with DNA. The whole transcriptome libraries were prepared using a Ribo-Zero Magnetic Gold kit (MRZE126, Zhongbeilinge Co., Ltd., Beijing, China) and a NEBNext RNA library preparation kit (E7775, New England Biolabs, Beverly, MA, USA). A BioAnalyzer 2100 system (Agilent Technologies, Palo Alto, CA, USA) was used for quality control and quantification. The obtained libraries were sequenced on a HiSeq2000 kit (Illumina Inc., San Diego, CA, USA) to analyze differentially expressed mRNAs. The volcano map for differentially expressed genes was produced using an R package (NIH, Bethesda, MA, USA). All procedures were conducted based on the manufacturer’s instructions [25].

### Reverse transcription quantitative polymerase chain reaction (RT-qPCR)

Total RNA from tissue homogenate or cells was extracted using Beyozol (R0011, Beyotime). Then, 1 μg RNA was reverse-transcribed to cDNA using an iScript cDNA synthesis kit (1708890EDU, Bio-Rad Inc., Hercules, CA, USA). Real-time qPCR was performed using a QuantiFast SYBR Green PCR kit (204054, Qiagen GmbH, Hilden, Germany) on Rotor-Gene 6000 (Corbett Research, Sydney, Australia). Relative expression of genes was examined using the 2^−ΔΔCT^ with GAPDH as the endogenous control [26]. The primers are shown in Table 1.

**Table 1.** Primers for RT-qPCR

Gene symbol	primer sequences (5’‐3’)
PTEN	F: AGAACGTGGGAGTAGACGGA
	R: CTTGACACTTGCAACCAGGC
SP1	F: CTCCAGACCATTAACCTCAGTGC
	R: CACCACCAGATCCATGAAGACC
GAPDH	F: CATCACTGCCACCCAGAAGACTG
	R: ATGCCAGTGAGCTTCCCGTTCAG

RT-qPCR, reverse transcription quantitative polymerase chain reaction; PTEN, phosphatase and tensin homolog; SP1, Sp1 transcription factor; GAPDH, glyceraldehyde-3-phosphate dehydrogenase

### Clinical samples

Seventy-two patients with T2DM-PTB and another 72 patients with T2DM alone treated at Shenyang Chest Hospital from December 2018 to December 2019 were included in the present study. Patients with a history of any blood borne diseases such as AIDs, hepatitis B, C, or other endocrine diseases were excluded. The blood samples were collected from patients to collect DNA and stored at −80°C. This study was approved by the Ethics Committee of Shenyang Chest Hospital (Approval Number: 2020–005-01) and strictly adhered to the *Declaration of Helsinki*. Signed informed consent was acquired from each respondent.

### Terminal deoxynucleotidyl transferase (TdT)-mediated dUTP nick end labeling (TUNEL)

The lung tissue slices were treated with 20 μg/mL proteinase K (3115836001, Roche Ltd., Basel, Switzerland) at 37°C for 15 min, and then blocked in the phosphate-buffered saline (PBS, 0.01% mol/L, pH = 7.4) at 37°C for 30 min. The slices were incubated with 50 μL TdT buffer at 37°C with 5% CO_2_ for 1 h and then reacted with 2% dUTP for 5 min. After that, the slices were treated with 50 μL blocker to block the nonspecific binding and then incubated with horseradish peroxidase (HRP)-labeled peroxidase (11428861001, Sigma-Aldrich, Merck KGaA, Darmstadt, Germany) at 37°C for 30 min. The slices were washed in PBS and developed in 3,3’-diaminobenzidine, and the nuclei were counter-stained with hematoxylin. The staining was observed under the microscope. Apoptotic cells were stained in brown. The apoptosis rate of cells was calculated by the percentage of brownish cells [27].

### Masson’s trichrome staining

The slices were dewaxed, rehydrated, stained with Weigert’s iron hematoxylin (G1142, Solarbio Science & Technology Co., Ltd., Beijing, China) for 2–10 min, differentiated in acidic ethanol differentiation solution, and returned to blue using the Masson’s solution. Thereafter, the slices were stained with ponceaus-fuchsin (P0012, Solarbio) for 5–10 min, stained with phosphomolybdic acid solution for 1 min, stained with aniline blue for 1 min, quickly dehydrated in 95% ethanol, and sealed with neutral balsam. Next, the slices were dehydrated in absolute ethanol for 3 times and viewed under a microscope (Olympus) to observe the portion of fibrotic tissues with 8 random fields of views included. The fibrotic areas were determined by the ImageProRPlus 6.0 software (Media Cybernetics, Bethesda, MD, USA) [28].

### Cell treatment and transfection

The macrophage RAW 264.7 cells were procured from American Type Culture Collection (Manassas, VA, USA) and cultured in Roswell Park Memorial Institute-1640 (R8758, Sigma-Aldrich) containing amino acid, 10% (v/v) fetal bovine serum and 1% penicillin/streptomycin. Expression of PTEN and SP1 in cells was altered through transfection of PTEN-OE, si-PTEN, si-SP1 or the controls PTEN-NC and SP1-NC (Sangon Biotech Co., Ltd., Shanghai, China). The cells were cultured in 6-well plates at 24 h prior to transfection. When the cell confluence reached 50%, the transfections were performed according to the instructions of Lipofectamine 2000 (11668030, Thermo Fisher Scientific Inc., Waltham, MA, USA). The cells were cultured in a 37°C incubator with 5% CO_2_ for 6 h, and then cultured in complete medium for 48 h before further use.

### Chromatin immunoprecipitation (ChIP)-qPCR

The upstream transcription factors of PTEN were predicted via JASPAR (http://jaspar.genereg.net/), and the binding sequence between SP1 and PTEN promoter was predicted using the ALGGEN system (http://alggen.lsi.upc.es/cgi-bin/promo_v3). Next, a ChIP kit (26157, Thermo Fisher Scientific) was used according to the manufacturer’s instructions. In short, 1 × 10^7^ cells were fixed in 1% methanol and quenched with glycine. The cells were lysed and separated in MNase (N3755, Sigma-Aldrich), and the mixtures were ultrasonicated to break the nuclear membrane. The cell lysates were incubated with anti-SP1 (ab231778, 1:100, Abcam Inc., Cambridge, MA, USA) or normal rabbit antibody immunoglobulin G (IgG, ab172730, 1:100, Abcam) at 4°C overnight. The DNA was diluted using ChIP dilution buffer, and the abundance of SP1 was examined by qPCR [29].

### Enzyme-linked immunosorbent assay (ELISA)

The murine lung tissues were minced and prepared as homogenate. The homogenate was centrifuged at 5,000 g for 5 min to collect the supernatant. The protein concentrations of interleukin-6 (IL-6), IL-8, IL-10 and C-X-C motif chemokine ligand 13 (CXCL13) were examined using a commercial ELISA kit (AmyJet Scientific Co., Ltd., Wuhan, Hubei, China) [26].

### Luciferase reporter gene assay

The promoter sequence of PTEN was obtained from UCSC (https://genome.ucsc.edu/index.html) and inserted to pGL3 vector (E171, Promega Corporation, Madison, WI, USA) to construct luciferase reporter vector. The vector was transfected in cells stably transfected with si-SP1. After 48 h, the luciferase activity was determined using the dual luciferase reporter system (Promega) [30].

### Western blot analysis

The murine lung tissue samples were made into homogenate and centrifuged at 20,000 at 4°C for 30 min to collect total protein, and the protein concentration was determined using the bicinchoninic acid method (23225, Pierce Biotechnology, Waltham, MA, USA). Then, the protein sample was separated by 10% sodium dodecyl sulfate-polyacrylamide gel electrophoresis and transferred onto polyvinylidene fluoride membranes. The membranes were incubated with the primary antibodies against SP1 (sc-420, 1:1,500, Santa Cruz Biotechnology, Inc, Santa Cruz, CA, USA), PTEN (MA5-12,278, 1:2,000, Thermo Fisher Scientific), AKT (sc-5298, 1:1,500, Santa Cruz), p-AKT (NB100-56749, 1:1,000, Novus Biologicals, Littleton, CO, USA) and GAPDH (ab8245, 1:2,000, Abcam) at 4°C for 16 h, and then with the secondary antibody (ab205719, 1:5,000, Abcam) at 37°C for 3 h. The protein bands were analyzed using an enhanced chemiluminescence (ECL) kit (PE0010, Solarbio) [31].

### Immunofluorescence staining

Cells were fixed, penetrated with 0.01% Triton X-100, and blocked with 5% goat serum for 30 min. After that, the cells were reacted with anti-PTEN (MA5-12278, 1:2,000, Thermo Fisher Scientific) and anti-SP1 (1:100, sc-420, Santa Cruz Biotechnology, Inc, Santa Cruz, CA, USA) overnight at 4°C, and then with Alexa Fluor® 488-labeled goat anti-mouse IgG (1:200, ab150113, Abcam) at 22–25°C for 1.5 h. The nuclei were counter stained by 4’, 6-diamidino-2-phenylindole. The cell slides were then sealed for observation under the fluorescence microscope [32].

### Immunohistochemistry (IHC)

Paraffin-embedded lung tissue sections were dewaxed in xylene, rehydrated in decreasing series of ethanol, treated with H_2_O_2_, added with sodium citrate buffer and heated in microwave oven. After that, the sections were blocked with 5% goat serum and reacted with anti-PTEN (1:50, GTX101025, GeneTex Inc., San Antonio, TX, USA), anti-SP1 (1:100, GTX1528, GeneTex), anti-CD86 (1:100, GTX32507, GeneTex) and anti-CD206 (1:100, PA5-101657, Thermo Fisher Scientific) at 4°C overnight, and then with the secondary antibody (1:1,000, ab6721, Abcam) at room temperature for 1 h. After 5 min of color development by 3,3’-diaminobenzidine, the nuclei were counter stained with hematoxylin, and then the section were sealed for microscopy observation [33].

### Statistical analysis

SPSS 22.0 (Corp. Armonk, NY, USA) was used for data analysis. Measurement data collected from three independent experiments were presented as the mean ± standard deviation. The *t* test was applied for data comparison between two groups, and one- or two-way analysis of variance (ANOVA) was applied for data comparison among multiple groups, followed by Tukey’s post-hoc test. The log rank test was used for post-statistical analysis. *p* was obtained from two-tailed test, *p* < 0.05 statistically significant difference was set at *p* < 0.05.

## Results

### Brief introduction

T2DM has been suggested to be linked to increased susceptibility to active TB. PTEN, which has been reported to be poorly expressed in diabetes, has been demonstrated to have functions in bacterial clearance. We established a murine model of T2DM, detected the PTEN expression in the model mice, and examined the exact correlation between T2DM and the PTB susceptibility. The candidate upstream regulator of PTEN was predicted by bioinformatics and validated by ChIP-qPCR and luciferase assays. Altered expression of PTEN and SP1 was induced in mice and in RAW264.7 macrophages to examine their functions in macrophage polarization and severity of MTB. The activity of Akt, a downstream target of PTEN was determined as well.

### Mice with T2DM have increased susceptibility to PTB

**Figure 1.**
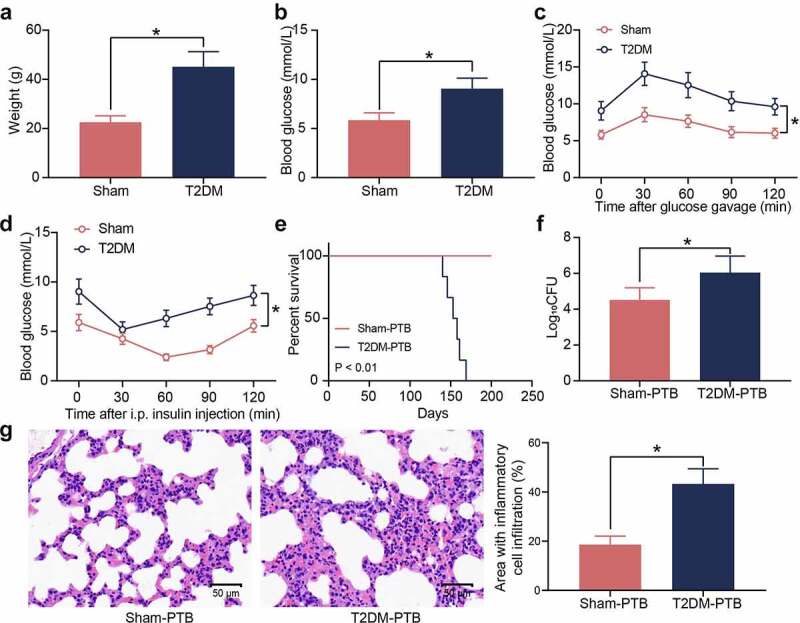
Mice with T2DM have increased susceptibility to PTB. A, weight of mice on the 8^th^ week after STZ injection (*p < 0.05, the unpaired *t* test); B, the blood glucose level in mouse examined by routine blood analysis (*p < 0.05, the unpaired *t* test); C, glucose tolerance of mice examined by the GTT (*p < 0.05, two-way ANOVA); D, insulin resistance of mice examined by the ITT (*p < 0.05, two-way ANOVA); E, survival days of mice after Mtb infection (*p* < 0.01, the Kaplan-Meier analysis); F, bacterial load in murine lung tissues examined by CFU analysis (*p < 0.05, the unpaired *t* test); G, infiltration of inflammatory cells in murine lung tissues examined by HE staining (*p < 0.05, the unpaired *t* test). For animal studies, n = 6 in each group.

T2DM is a heterogeneous disease featured with metabolic dysregulation of blood glucose and lipids. A murine model of T2DM was first established by STZ injection. Compared to the sham-operated mice, the mice with T2DM had significant increases in body weight (Figure 1(a)) and blood glucose levels (Figure 1(b)). The GTT results showed that the STZ-treated mice showed significant glucose intolerance (Figure 1(c)), and the ITT results indicated that these mice showed significant insulin resistance after insulin injection (Figure 1(d)). The T2DM mice and the sham-operated mice were treated with Mtb H37Rv via aerosol infection. The survival time of mice was set at 200 days. From the 140 days post infection (dpi), the diabetic mice showed PTB symptoms, and their survive rate was decreased, whereas all the sham-operated mice survived during the whole process (Figure 1(e)). The bacterial load in murine lung tissues after animal death was examined. Significantly increased bacterial load was observed in the lung tissues of mice with T2DM (figure 1(f)). Also, the HE staining results showed that the mice with T2DM had significantly increased inflammatory cell infiltration in their lung tissues (Figure 1(g)). These results suggest that the mice with T2DM possibly have increased susceptibility to Mtb infection and aggravated lung injury after infection.

### PTEN downregulation is possibly correlated with the susceptibility to PTB

**Figure 2.**
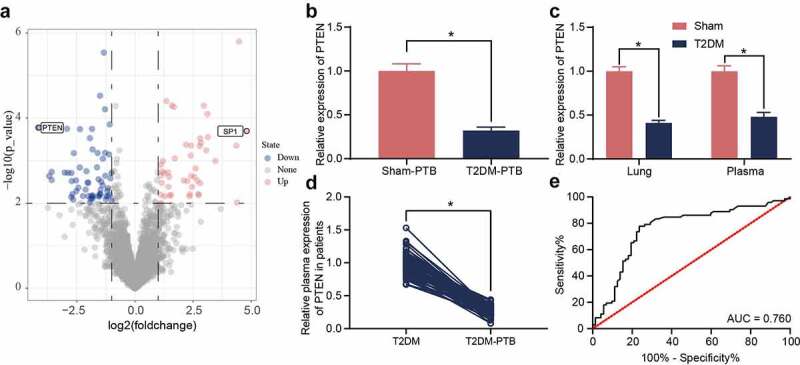
PTEN downregulation is possibly correlated with the susceptibility to PTB. A, differentially expressed mRNAs between the lung tissues from mice with T2DM and the sham-operated ones identified by mRNA microarray analysis; B-C, PTEN expression in murine lung tissues determined by RT-qPCR (*p < 0.05, the unpaired *t* test); D, PTEN expression in plasma of clinical patients examined by RT-qPCR (n = 72, *p < 0.05, the unpaired *t* test); E, specificity of PTEN expression to the susceptibility to T2DM-PTB analyzed by a ROC and the AUC (*p < 0.05, the Wilson/Brown analysis). For animal studies, n = 6 in each group.

The diabetic mice and sham-operated mice were infected with Mtb, and then the murine lung tissues were collected for mRNA microarray analysis. A total of 46 upregulated mRNAs whereas 68 downregulated mRNAs were identified (Figure 2(a)). PTEN was found to be downregulated in the tissues of mice with T2DM-PTB (Figure 2(b)). In addition, PTEN was downregulated in the plasma as well as lung tissues of mice with T2DM even before Mtb infection, indicating that the PTEN downregulation might be correlated with the susceptibility of mice to PTB (Figure 2(c)). The PTEN level in plasma of patients was determined. It was observed that the PTEN expression was decreased in patients with T2DM-PTB compared to those with T2DM only (Figure 2(d)). A receiver operating characteristic (ROC) curve was produced to examine the correlation between PTEN expression and the susceptibility of patients to PTB. The area under curve (AUC) was 0.760, indicating PTEN as a potential resistant gene of diabetic patients to PTB (Figure 2(e)).

### Overexpression of PTEN enhances the Mtb resistance of mice with T2DM

**Figure 3.**
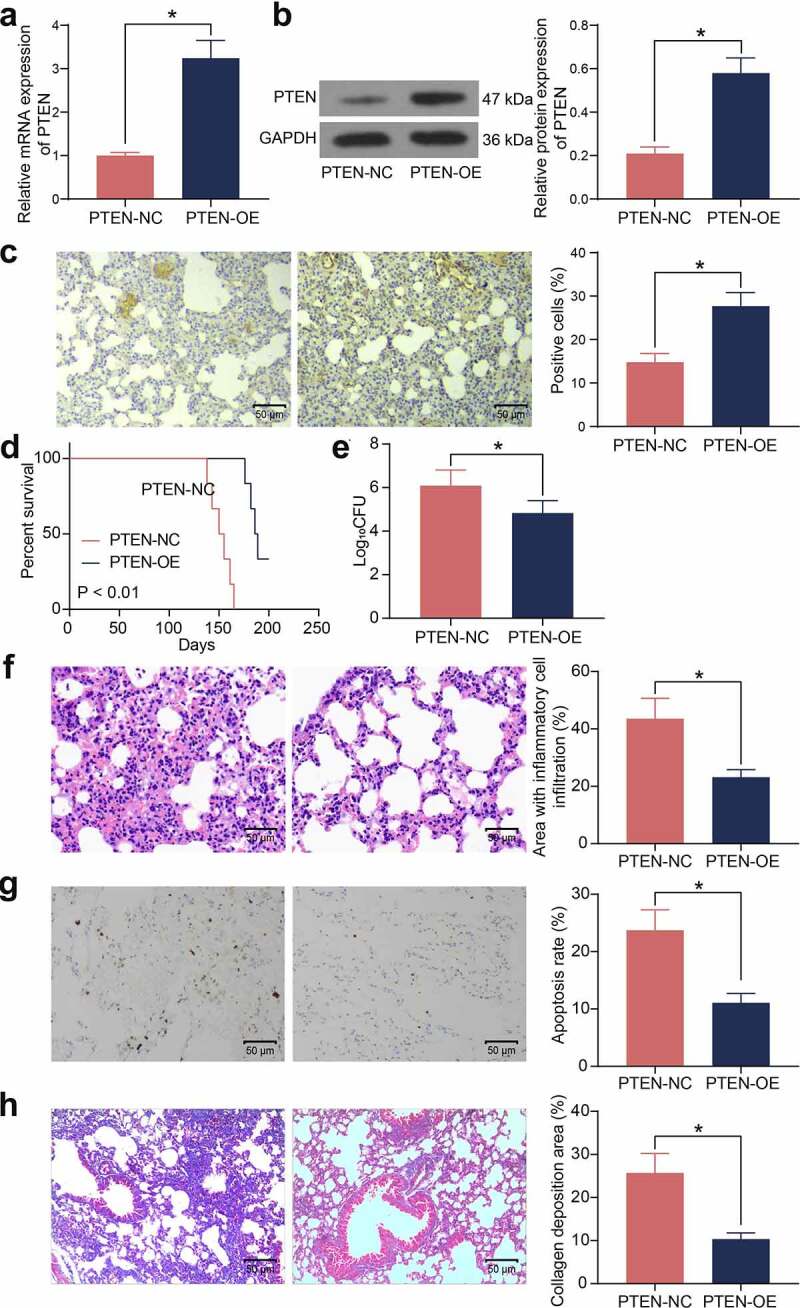
Overexpression of PTEN enhances the Mtb resistance of mice with T2DM. A-C, PTEN expression in murine lung tissues examined by RT-qPCR (a), western blot analysis (b), and IHC (c) (*p < 0.05, the unpaired *t* test); D, survival days of the mice after PTEN-OE administration (*p* < 0.01, the Kaplan-Meier analysis); E, bacterial load in murine lung tissues examined by CFU analysis (*p < 0.05, the unpaired *t* test); F, infiltration of inflammatory cells in murine lung tissues examined by HE staining (*p < 0.05, the unpaired *t* test); G, epithelial cell apoptosis in the murine lung tissues after Mtb infection examined by the TUNEL assay (*p < 0.05, the unpaired *t* test); H, fibrosis in murine lung tissues determined by Masson’s trichrome staining. For animal studies, n = 6 in each group.

To validate the role of PTEN in the susceptibility to Mtb infection, overexpression of PTEN was induced in mice with T2DM, and the stable upregulation of PTEN was detected in the lung tissues by RT-qPCR, western blot analysis, and IHC (Figure 3(a-c)). In addition, mice administrated with PTEN-OE showed significantly improved survival rate during the long-term Mtb infection process (Figure 3(d)). Also, overexpression of PTEN significantly reduced the bacterial load in the murine lung tissues after Mtb infection (Figure 3(e)). The HE staining suggested that overexpression of PTEN reduced the inflammatory area as well as the infiltration of inflammatory cells in the murine lung tissues (figure 3(f)). Moreover, the number of apoptotic epithelial cells in the murine lung tissues after Mtb infection, according to the TUNEL assay, was significantly reduced when PTEN was upregulated (Figure 3(g)). The Masson’s trichrome staining suggested that overexpression of PTEN also significantly reduced the number of fibrotic nodules in the murine lung tissues (Figure 3(h)).

### SP1 suppresses PTEN transcription

**Figure 4.**
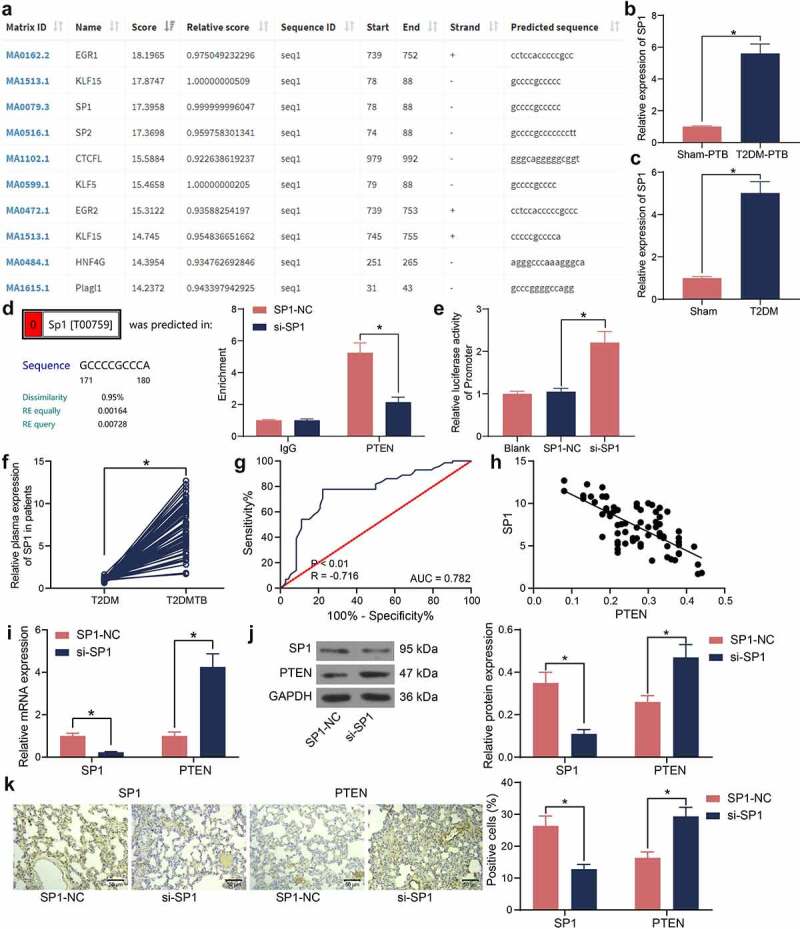
SP1 suppresses PTEN transcription. A, upstream transcription factors of PTEN predicted on JASPAR; B-C, SP1 expression in lung tissues of mice with T2DM-PTB (b) or T2DM only (c) examined by RT-qPCR (*p < 0.05, the unpaired *t* test); D, binding relationship between SP1 and PTEN examined by the ChIP-qPCR assay (*p < 0.05, two-way ANOVA); E, binding relationship between SP1 and PTEN promoter examined by luciferase reporter gene assay; F, SP1 expression in lung tissues of patients with T2DM or T2DM-PTB (n = 72, *p < 0.05, the unpaired *t* test); G, specificity of SP1 expression to the susceptibility to T2DM-PTB analyzed by a ROC and the AUC (*p < 0.05, the Wilson/Brown analysis); H, an inverse correlation between SP1 and PTEN expression according to Pearson’s correlation analysis; I, PTEN expression in murine lung tissues in the setting of SP1 silencing (*p < 0.05, the two-way ANOVA); J, protein levels of SP1 and PTEN in murine lung tissues determined by western blot analysis (*p < 0.05, the two-way ANOVA); K, SP1 and PTEN expression in lung tissues examined by IHC assay (*p < 0.05, the two-way ANOVA). For animal studies, n = 6 in each group.

The upstream transcription factors of PTEN were predicted using the JASPAR system, and the top 10 transcription factors with the highest binding score with PTEN promoter was selected. SP1 was suggested to have a high binding degree with PTEN (Figure 4(a)). Of note, SP1 was identified as one of the significantly increased mRNAs in the lung tissues of mice with T2DM-PTB in Figure 2(a). Therefore, the SP1 expression in the murine tissues was examined by RT-qPCR. It was found that the SP1 expression was significantly increased in mice with T2DM-PTB or with T2DM only compared to the sham-operated mice (Figure 4(b,c)). The binding between SP1 and PTEN was validated in the RAW 264.7 cells using the ChIP-qPCR assay. It was observed that silencing of SP1 decreased the SP1 fragments enriched by PTEN promoter (Figure 4(d)). The luciferase assay also revealed that silencing of SP1 increased the luciferase activity of the PTEN reporter vector in cells (Figure 4(e)). Also, increased expression of SP1 was detected in the plasma of patients with T2DM-PTB compared to those with T2DM only (figure 4(f)). The ROC curve indicated SP1 showed specific judging value in the Mtb susceptibility of patients with T2DM (Figure 4(g)). An inverse correlation between SP1 and PTEN was identified in the plasma of patients with T2DM-PTB (Figure 4(h)). Lentiviral vector-carried si-SP1 was injected into mice, and then RT-qPCR, western blot analysis, and IHC results revealed that the SP1 expression in murine lung tissues was successfully suppressed. In this setting, the mRNA and protein levels of PTEN were significantly elevated (Figure 4(i-k)). These results indicated that downregulation of SP1 is possibly correlated with the development of PTB.

### SP1/PTEN mediates macrophage polarization in mice with T2DM-PTB

**Figure 5.**
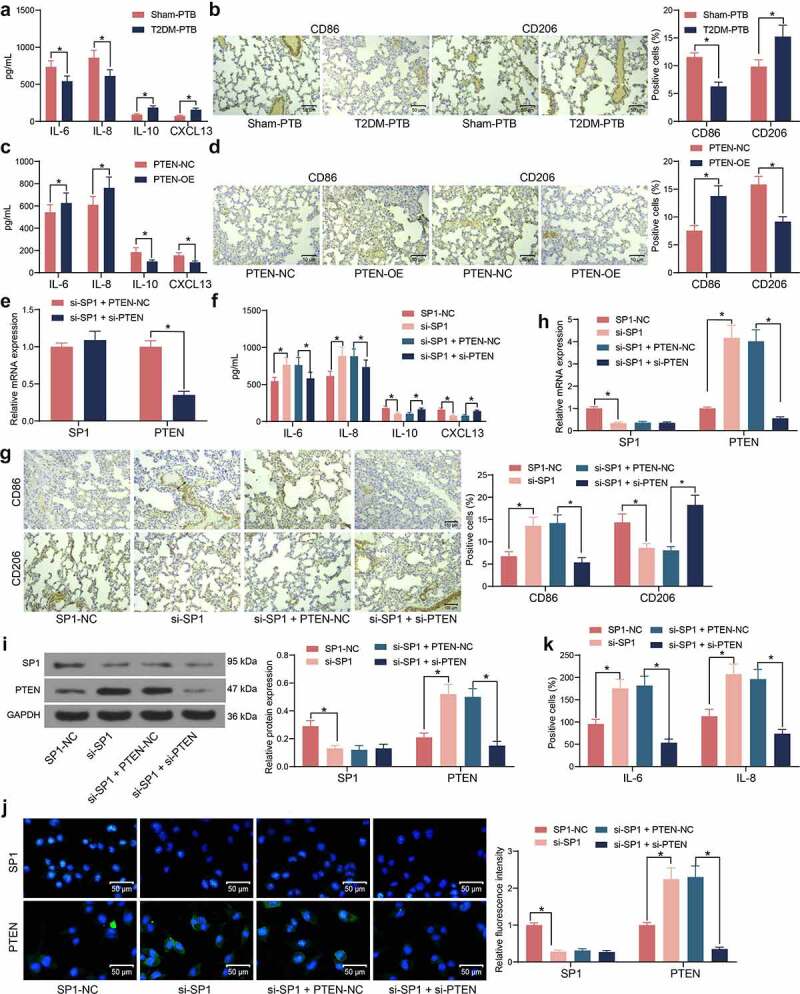
SP1/PTEN mediates macrophage polarization in mice with T2DM-PTB. A, concentrations of the M1 macrophage-specific cytokines IL-6 and IL-8 and the M2 macrophage-specific cytokines IL-10 and CXCL13 in murine lung tissues determined using ELISA kits (*p < 0.05, two-way ANOVA); B, expression of macrophage markers CD86 (M1) and CD208 (M2) in murine lung tissues determined by IHC (*p < 0.05, two-way ANOVA); C, concentrations of IL-6, IL-8, IL-10 and CXCL13 in murine lung tissues after PTEN-OE administration determined using ELISA kits (*p < 0.05, two-way ANOVA); D, expression of macrophage markers CD86 (M1) and CD208 (M2) in murine lung tissues after PTEN-OE administration determined using IHC (*p < 0.05, two-way ANOVA); E, expression of PTEN in lung tissues after si-SP1 + si-PTEN administration examined by RT-qPCR (*p < 0.05, two-way ANOVA); F, expression of the levels of IL-6, IL-8, IL-10 and CXCL13 in murine lung tissues after si-SP1 and si-PTEN administration examined using ELISA kits (*p < 0.05, two-way ANOVA); G, expression of macrophage markers CD86 (M1) and CD208 (M2) in murine lung tissues after si-SP1 and si-PTEN administration determined using IHC (*p < 0.05, two-way ANOVA); H-J, expression of SP1 and PTEN in RAW 264.7 cells after si-SP1 and si-PTEN administration examined by RT-qPCR, western blot analysis, and immunofluorescence staining (*p < 0.05, two-way ANOVA); K, concentrations of IL-6, IL-8 in the supernatant of RAW 264.7 cells determined using ELISA kits (*p < 0.05, two-way ANOVA).

To determine the role of the SP1/PTEN axis in PTB, the phenotype changes in macrophages in murine lung tissues during the process of Mtb infection were explored. The ELISA kits showed that the concentrations of M1 macrophage-specific cytokines IL-6 and IL-8 were lower whereas the concentrations of M2 macrophage-specific cytokines IL-10 and CXCL13 were higher in T2DM-PTB mice than those in the Sham-PTB mice (Figure 5(a)). Moreover, we observed that the expression of the M1 macrophage marker CD86 was decreased whereas the M2 marker CD206 was increased in tissues of T2DM-PTB mice (Figure 5(b)), indicating that T2DM might promote the phenotype transition from M1 to M2 in murine lung tissues to enhance the susceptibility to PTB. Of note, overexpression of PTEN in mice increased the IL-6 and IL-8 levels and CD86 expression but reduced the IL-10 and CXCL13 levels and CD206 expression in their lung tissues (Figure 5(c,d)), suggesting that PTEN might lead to M1 polarization of the macrophages. After that, si-SP1 was administrated into mice alone or concomitant with si-PTEN (Figure 5(e)). Silencing of SP1 significantly upregulated the IL-6 and IL-8 levels and CD86 expression whereas reduced the IL-10 and CXCL13 levels and CD206 expression in the lung tissues, but further silencing of PTEN inversed these changes (figure 5(f,g)). *In vitro*, si-SP1 and si-PTEN were induced in RAW264.7 cells. The RT-qPCR, western blot and immunofluorescence staining results showed that silencing of SP1 significantly increased the PTEN expression in cells, which was blocked by si-PTEN (Figure 5(h-j)). The ELISA results showed that the IL-6 and IL-8 levels in cells were elevated upon SP1 silencing but decreased after PTEN suppression (Figure 5(k)). Therefore, we opine that the SP1/PTEN regulates macrophage polarization to affect the survival of Mtb.

### SP1/PTEN mediates lung infection in mice and the activity of the Akt signaling pathway

**Figure 6.**
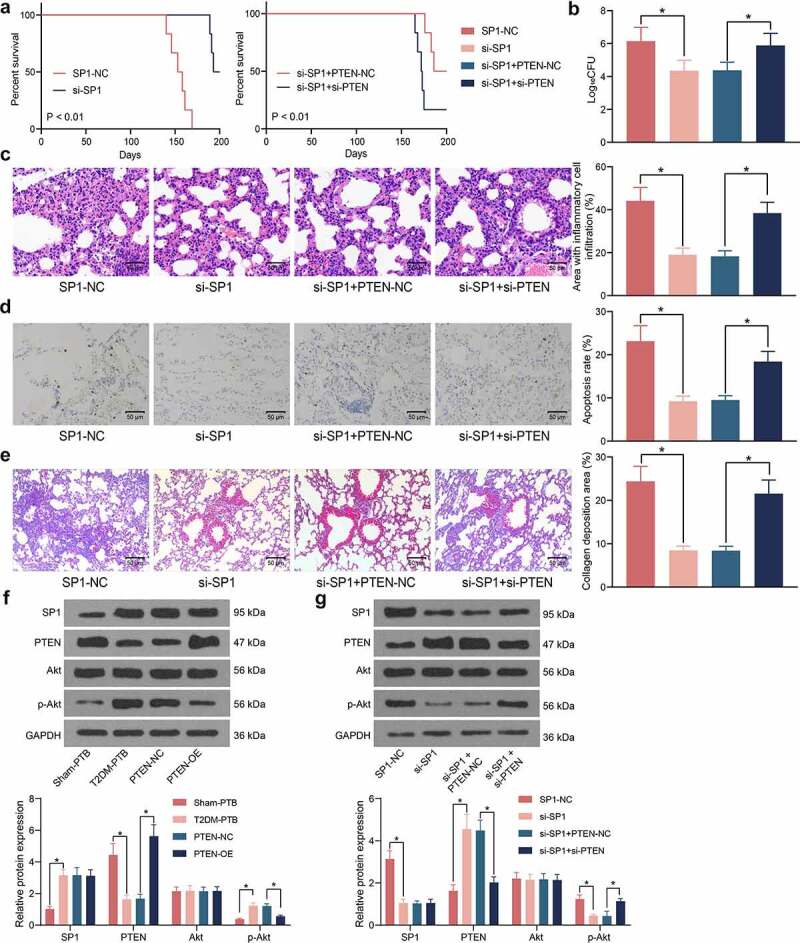
SP1/PTEN mediates lung infection in mice and the activity of the Akt signaling pathway. A, survival days of the mice after si-SP1 and si-PTEN administration (*p* < 0.01, the Kaplan-Meier analysis); B, bacterial load in murine lung tissues examined by CFU analysis (*p < 0.05, the one-way ANOVA); C, infiltration of inflammatory cells in murine lung tissues examined by HE staining (*p < 0.05, the one-way ANOVA); D, epithelial cell apoptosis in the murine lung tissues after Mtb infection examined by the TUNEL assay (*p < 0.05, the one-way ANOVA); E, fibrosis in murine lung tissues determined by Masson’s trichrome staining (*p < 0.05, the one-way ANOVA); F-G, protein levels of SP1 and PTEN and the Akt phosphorylation in murine lung tissues determined by western blot analysis (*p < 0.05, the one-way ANOVA). For animal studies, n = 6 in each group.

The role of SP1 in the mouse survival following Mtb infection was examined. It was observed that silencing of SP1 significantly increased the survival rate of the mice with T2DM-PTB, and further silencing of PTEN blocked the role of si-SP1 and reduced the survival rate of animals (Figure 6(a)). Moreover, the bacterial load in murine lung tissues was significantly reduced by si-SP1 but then restored by si-PTEN (Figure 6(b)). The HE staining results indicated that SP1 silencing alleviated the inflammatory responses in the lung tissues, whereas the infiltration of inflammatory cells in the tissues was significantly increased following additional PTEN silencing (Figure 6(c)). TUNEL results indicated that SP1 downregulation also significantly reduced the apoptosis of epithelial cells, whereas PTEN knockdown restored the cell apoptosis in the lung tissues (Figure 6(d)). The Masson’s trichrome staining also indicated that si-SP1 reduced the tissue fibrosis in the mice with T2DM-PTB, whereas si-PTEN increased the degree of tissue fibrosis again (Figure 6(e)). As PTEN is a key gene on the Akt signaling pathway, we surmised that SP1 possibly regulates the PTEN/Akt signaling to induce the lung injury in mice with T2DM-PTB. Thereafter, the levels of SP1 and PTEN and the Akt phosphorylation in murine lung tissues was examined. Importantly, the mice with T2DM-PTB had increased protein level of SP1, reduced level of PTEN, and increased phosphorylation of Akt compared to the Sham-PTB mice, and silencing of PTEN significantly elevated the Akt phosphorylation in mice (figure 6(f)). Downregulation of SP1 in mice led to an increase in the protein level of PTEN whereas a decline in Akt phosphorylation, but further silencing of PTEN restored the Akt phosphorylation in the murine tissues (Figure 6(g)). Therefore, it can be concluded that the SP1 possibly mediates lung infection in mice through the PTEN/Akt axis.

## Discussion

Approximately over a million TB cases were affected by DM in 2012 alone [34], while the number of adult DM cases is projected to rise from 463 million in 2019 to 578 million by 2030 and 700 million by 2045 worldwide (https://diabetesatlas.org/en/), indicating a foreseeable increase in the incidence of T2DM-associated TB as well. Although there are effective drugs against Mtb infection through treatment with combined 3–4 drugs for continuous 8–9 months, there is neither effective vaccines nor biomarkers for protective immunity [35]. In the present study, PTEN downregulation by SP1 was identified as a susceptible factor to Mtb infection in T2DM-PTB, which was possibly induced by the Akt activation and M2 polarization of macrophages.

A murine model of T2DM was first established, and these mice showed significantly increased weight, blood glucose level, glucose intolerance and insulin resistance, and as expected, increased susceptibility to PTB following Mtb infection since T2DM individuals are well known susceptible to a various of bacterial infections [4]. Of note, the mRNA microarray analysis on lung tissues of mice identified that PTEN was significantly downregulated in mice with T2DM-PTB relative to those with T2DM before Mtb infection, and downregulation of PTEN was identified in the serum sample of patients with T2DM-PTB compared to those with T2DM only. These results indicated that the PTEN downregulation might confer susceptibility to Mtb infection in individuals. PTEN has been documented to play a key role in resisting infections by *bacillus Calmette-Guérin* and Mycoplasma in mammalian cells [21]. In a work by Lu *et al*., PTEN was identified as one of the four key factors mediated by the differentially expressed microRNAs between patients with and without latent tuberculosis infections [36]. However, the specific role of PTEN in Mtb infection remains unexplained. In the present study, artificial upregulation of PTEN significantly elevated the survival rate of mice following Mtb infection, reduced bacterial load, inflammatory infiltration, cell apoptosis, and fibrosis in lung tissues. These results identified PTEN plays an important role in Mtb resistance, and PTEN downregulation might be correlated with the susceptibility of individuals to Mtb infection.

Thereafter, the bioinformatics analysis suggested SP1 as an important upstream regulator of PTEN. The binding between SP1 and PTEN promoter was then validated via ChIP-qPCR and luciferase assays, and increased SP1 expression was identified in the lung tissues of mice as well as the serum sample of patients with T2DM-PTB. Intriguingly, SP1 has been proposed as a key molecule affecting the hub genes implicated in the common mechanisms between T2DM and Parkinson’s disease [37]. The expression and mitochondrial fission of SP1 was found to be significantly increased in a diabetic or high-glucose environment [38]. Similarly, elevated SP1 level was detected in diabetic submandibular glands as well as the high-glucose-treated SMG-C6 cells, which was induced by the downregulation of its suppressor microRNA-22-3p [39]. As a transcription factor, SP1 bound to the promoter of phosphoglycerate mutase family member 5 to aggravate diabetic renal tubular injury [38]. Inhibition of SP1 led to a decline in the transcription of tryptophan-aspartate containing coat protein, which increased maturation of phagosomes within macrophages to suppress Mtb survival [40]. Following MTB infection, the issue resident alveolar macrophages are biased toward an M2-like phenotype, which are more permissive to bacterial growth than the pro-inflammatory, monocyte-derived M1-like macrophages [35]. Proinflammatory cytokines, including interleukins (IL)-12, IL-23, IL-18, and tumor necrosis factor-α (TNF-α) produced by infected macrophages and dendritic cells can induce the differentiation of Th1 cell and production of Th1 and Th1-like cytokines, such as interferon-γ (IFN-γ) and IL-2 which play critical roles in the expression of host resistance against mycobacterial infections [14,15]. By contrast, immunosuppressive cytokines such as IL-4, IL-10 and TGF-β produced by M2 macrophages are associated with immunodeficiency and the persistence and advanced infection with pathogens including MTB [15,41]. After successful treatment of PTB in infected patients, the type 2 inflammatory environment shifts back to type 1 [14,42]. Moreover, elevation of M1 macrophages has been observed in non-diabetic mice subjected *Bacillus Calmette-Guérin* vaccine, a vaccine against Mtb infection, whereas mice with T2DM showed increased M2 macrophages compared to the non-diabetic mice [43]. Likewise, patients with T2DM-PTB were found to have increased IL10 concentrations compared to non-diabetic patients and vaccinated individuals [44]. Of note, we found that PTEN overexpression or SP1 silencing elevated the expression of M1 phenotype cytokines and markers (IL-6, IL-8 and CD86) whereas reduced the expression of M2 phenotype biomarkers (IL10, CXCL13 and CD206), along with reduced bacterial load in the murine lung tissues. During this process, the Akt phosphorylation was reduced by SP1 silencing but recovered after further PTEN silencing. Activation of Akt has been reported to be required for the adipose tissue inflammation and cell hypertrophy in TB [45]. The Akt signaling has been documented to play a considerable role in activation and polarization of macrophages [46]. Akt exerts key functions in infectious diseases and innate immunity, including the regulation of functional activation of macrophages [47,48]. Moreover, Akt activation is essential for M2 activation since Akt inhibition diminishes the elevation of M2 genes [49–51]. On the contrary, deletion of PTEN leads to Arg1 upregulation and M2 polarization of macrophages [52,53]. Overall, regulation of the PI3K/Akt signaling is a central node in the control of macrophage polarization.

## Conclusion

In summary, this paper demonstrates that downregulation of PTEN, which is induced by the elevated levels of SP1 in T2DM, predisposes individuals to Mtb infection by inducing the M2 polarization of macrophages via activation of Akt. However, due to the time and financial limitations, we did not include another group of model mouse with T2DM-MTB to investigate the exact roles of Akt in macrophage polarization. We would like to administrate Akt-specific inhibitors or activators in mice to further validate the implication of this signaling in the SP1/PTEN-regulated macrophage polarization and the severity of PTB. Anyway, this study may offer new insights into the field that PTEN downregulation might confer susceptibility to Mtb and therefore it might serve as a biomarker for protective immunity in T2DM-PTB.

## Supplementary Material

Supplemental Material

## Data Availability

The data used to support the findings of this study are included within the article.
