# Supplementary material for: Sp1 transcription factor represses transcription of phosphatase and tensin homolog to aggravate lung injury in mice with type 2 diabetes mellitus-pulmonary tuberculosis
Source: Bioengineered. 2022 Apr 14;13(4):9928–44. doi: 10.1080/21655979.2022.2062196 (PMC9162029; doi:10.1080/21655979.2022.2062196)
Supplement: Supplemental Material [file KBIE_A_2062196_SM4886.zip › supplementary/ethical 1.pdf]

沈阳市胸科医院动物实验伦理审查表

Ethical Review Form of Animal Experiment in Shenyang Chest Hospital

一、项目与人员信息

课题名称 (Project Title)	SP1抑制PTEN转录激活Akt通路对2型糖尿病并发肺结核感染的影响		
课题负责人 Name of principal Investigator	赵红梅 Hongmei Zhao		
单位/科室 Department	结核内科 Department of Tuberculosis	信箱 E-mail	Zhaohongmei2271@163.com
课题参与者 Project participants	赵红梅，石莲，王晓虹，于秀丽，王丹凤		

二、实验动物信息

动物来源 Animal origin	√ □采购/赠予(Procurement / Gift)（具体单位名称：SLAC实验室动物有限公司	质量合格证 Certification of fitness	☑ 有Yes<br>☐ 无No
品种/品系 breed/strain	C57BL / 6J小鼠		
数量（只） Number 60 只		周/月龄 W/M Age	20±5 g/4周龄
拟实验时间：Proposed Experimental period	2020年2月-2021年3月		

三、 研究项目信息

声明: 1. 我将自觉遵守实验动物福利伦理相关法规和各项规定，同意接受伦理委员会和实验动物室管理者的监督与检查；

2. 本人保证本申请表中所填内容真实、详尽和易懂。

Declaration：

1. I will abide by the law and regulation stipulation, and accept the supervision and inspection by the committee and laboratory animal department.
2. The information I have given is accurate, detailed and comprehensive.

声明人：课题负责人签字 Declarant：Signature of PI 赵红梅

伦理委员会审批意见 Approval opinion：

√ □ 批准 Approval　　□ 不批准 Not approve

指定负责人签（章）：

Authorized Personnel Signature（Seal）

2020年01月15日
