# Supplementary material for: Sp1 transcription factor represses transcription of phosphatase and tensin homolog to aggravate lung injury in mice with type 2 diabetes mellitus-pulmonary tuberculosis
Source: Bioengineered. 2022 Apr 14;13(4):9928–44. doi: 10.1080/21655979.2022.2062196 (PMC9162029; doi:10.1080/21655979.2022.2062196)
Supplement: Supplemental Material [file KBIE_A_2062196_SM4886.zip › supplementary/ethical 2.pdf]

沈阳市胸科医院伦理审查表

(Ethics Review Form of Shenyang Chest Hospital)

1.课题题目 (Project Title):

SP1 抑制 PTEN 转录激活 Akt 通路对 2 型糖尿病并发肺结核感染的影响

2.基本信息 (Basic Info):

研究负责人 (Principle Investigator):

姓名 (Name): 赵红梅 Hongmei Zhao

邮件 (E-MAIL): Zhaohongmei2271@163.com

拟研究时间 (Proposed Research Time): 2020 年 2 月-2021 年 3 月

3. 研究负责人的保证书 (Certification of Principle Investigator):

本人声明所填写内容属实，并将严格按照申请书中有关内容从事实验和研究。本人表示严格遵守国家法律和实验室有关规定，同时保护受试人的健康、权益和隐私。在实施过程中发生严重不良反应或不良事件的，本人有责任向沈阳市胸科医院如实报告。

My signature below certifies that the research described in this application and supporting materials will be conducted in full compliance with government regulations and laboratory's policies, especially those governing human subjects research. I will promptly report any unanticipated problems or adverse events to Shenyang Chest Hospital.

研究负责人签名 Signature of Principle Investigator (PI) 赵红梅

4. 伦理审查委员会意见 (Opinion of the Scientific Review Committee):

对该研究申请人主持此项工作的意见 (Opinion of the Competency of the Investigator(s) to Conduct this Project):

同意 Agree（√） 不同意 Disagree ( )

以下印章确认本委员会已经考察了研究申请人的科研水平和所提科研项目的科研价值，并同意该研究申请人主持此项目研究工作。

The seal below certifies that I have reviewed this research protocol and that I approve the investigator(s) to conduct this project in this laboratory.

印章（Seal）

沈阳市胸科医院 伦理委员会专用章

Date: 2020.1.11
