# Supplementary figures and images for: Sp1 transcription factor represses transcription of phosphatase and tensin homolog to aggravate lung injury in mice with type 2 diabetes mellitus-pulmonary tuberculosis
Source: Bioengineered. 2022 Apr 14;13(4):9928–44. doi: 10.1080/21655979.2022.2062196 (PMC9162029; doi:10.1080/21655979.2022.2062196)

**Original full-size western blot images**

**Fig 3B**

**
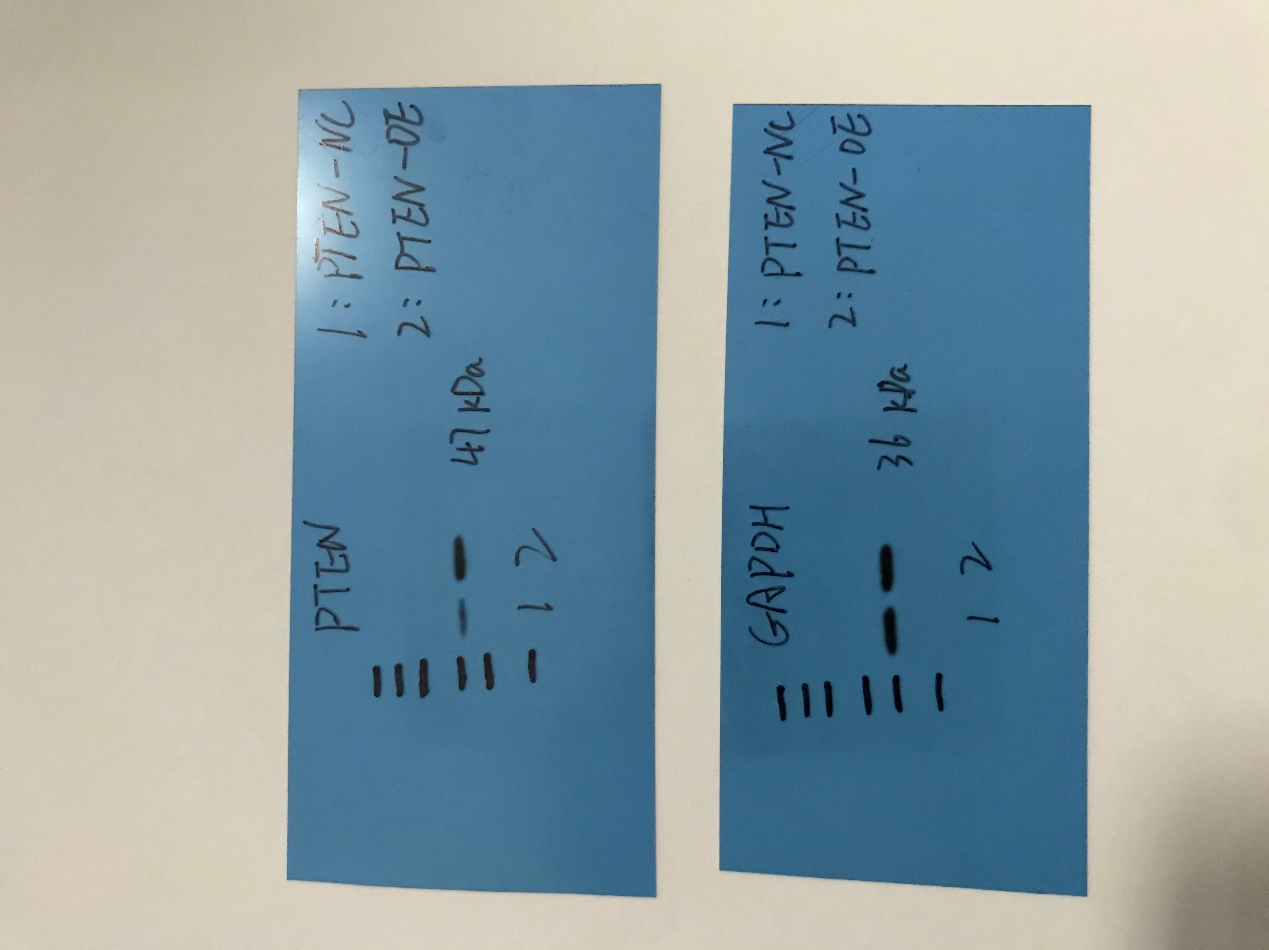
**

**Fig 4J**

**
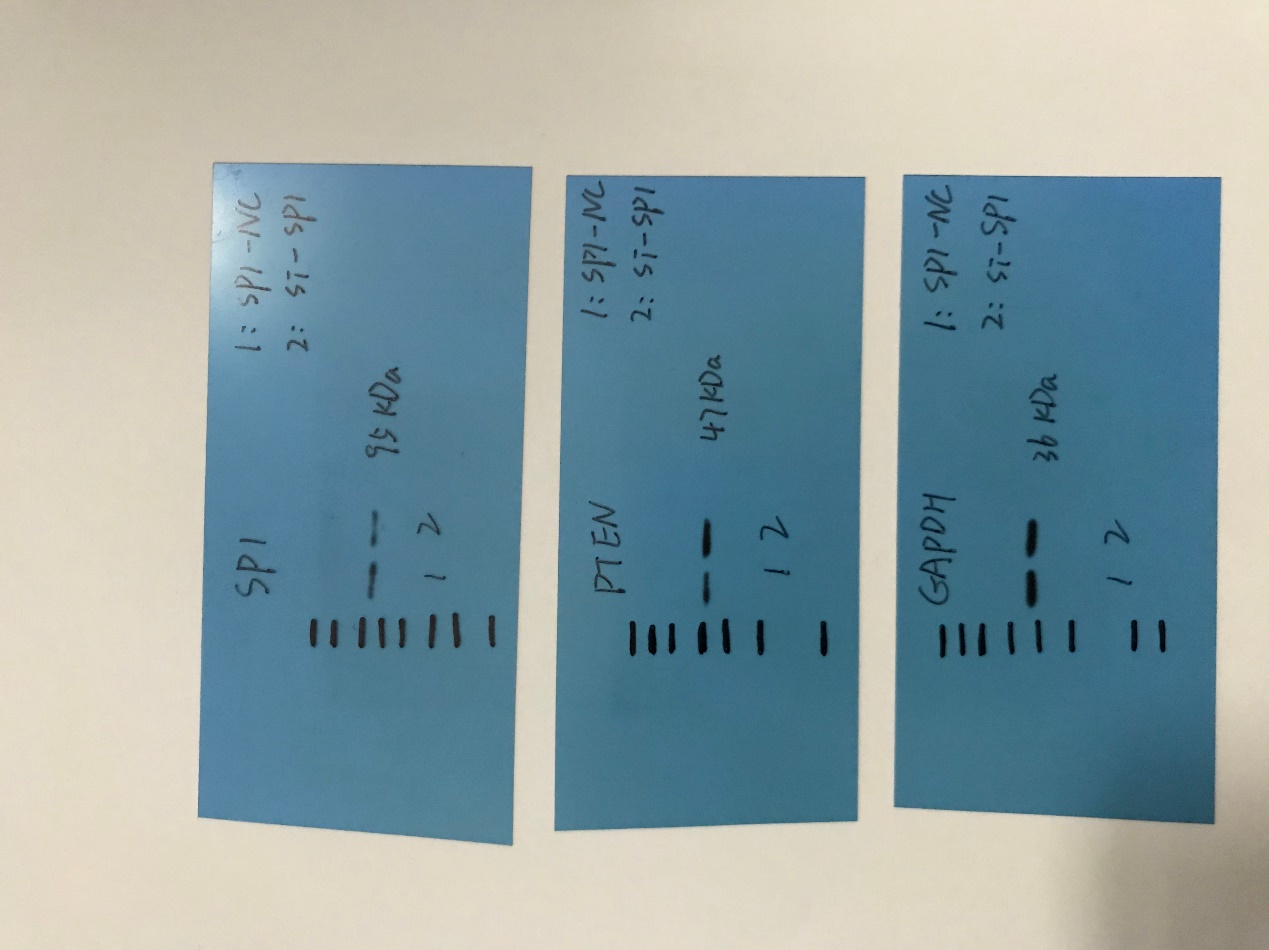
**

**Fig 5I**

**
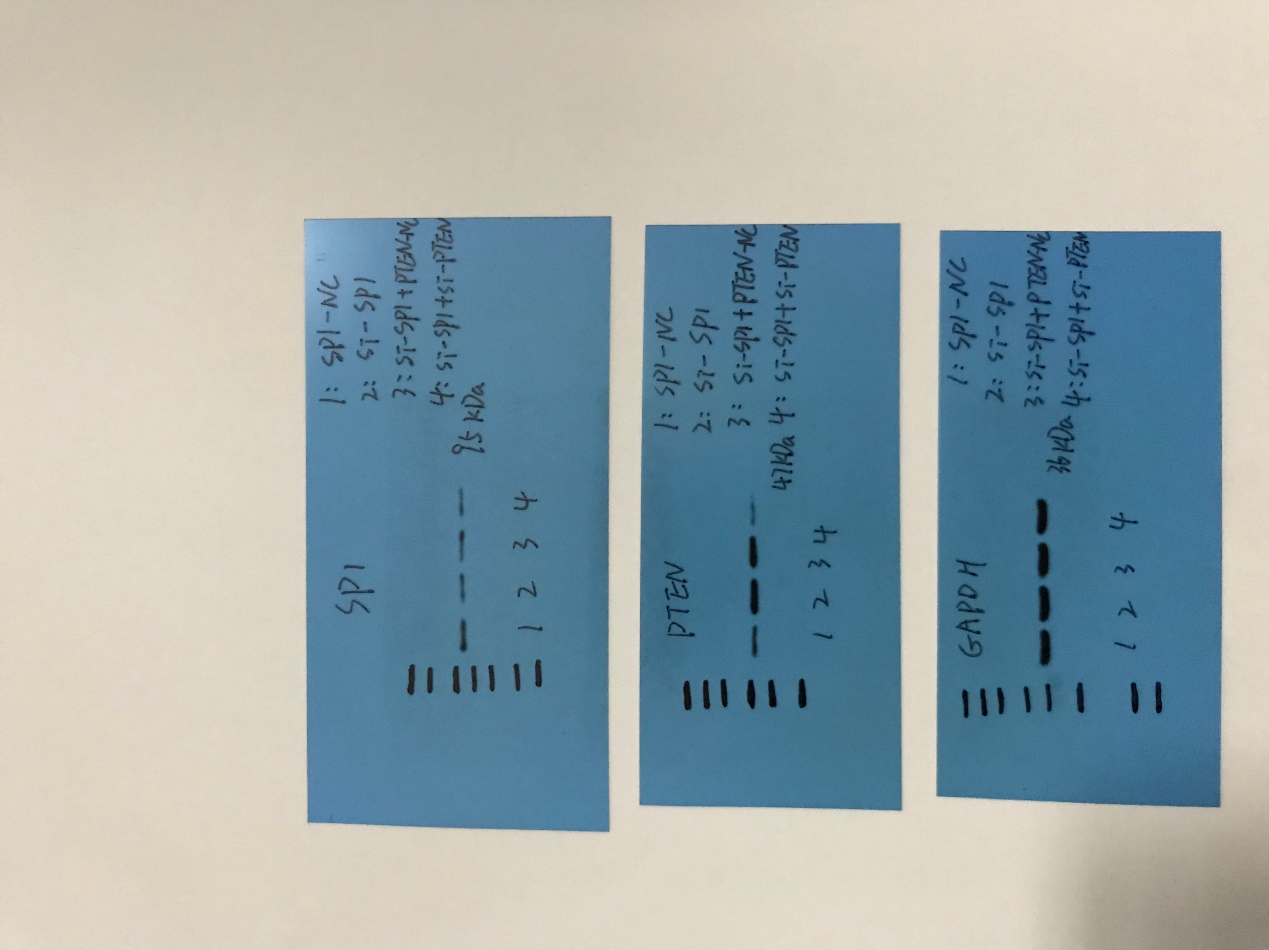
**

**Fig 6F**

**
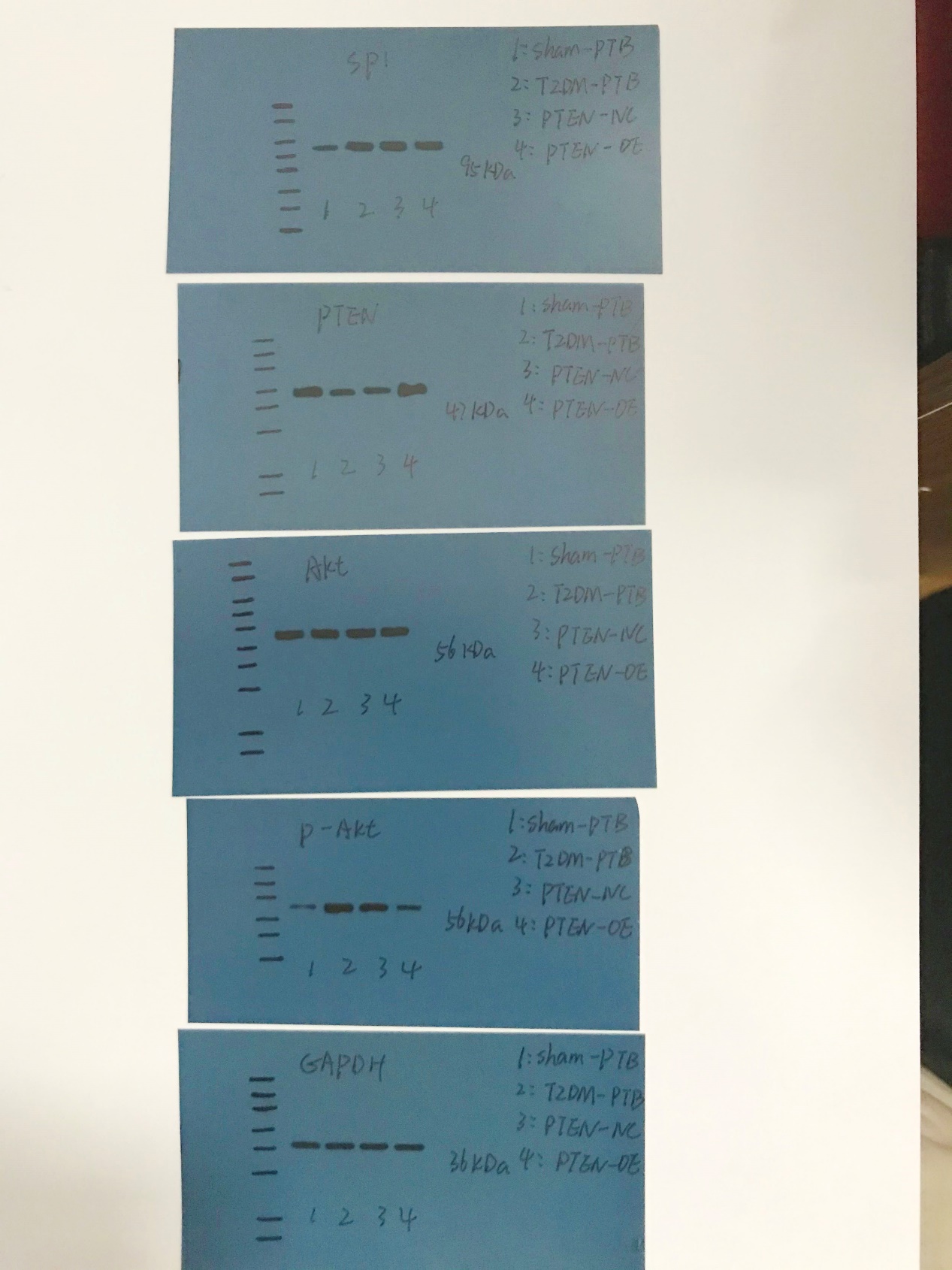
**

**Fig 6G**

**
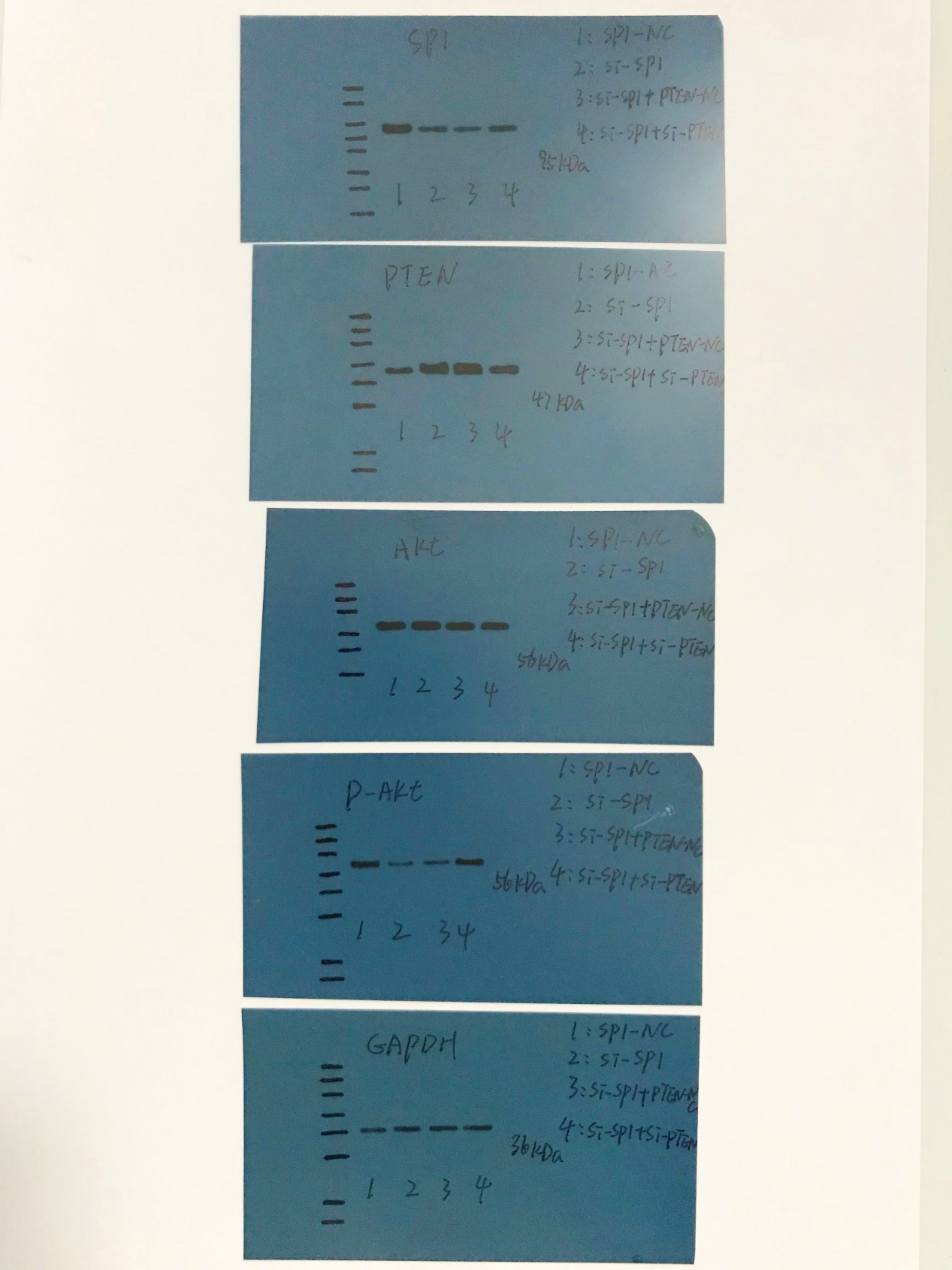
**

Supplement: Supplemental Material [file KBIE_A_2062196_SM4886.zip › supplementary/Original full size western blot images.docx]
